# Preclinical efficacy of CIGB-300, an anti-CK2 peptide, on breast cancer metastasic colonization

**DOI:** 10.1038/s41598-020-71854-6

**Published:** 2020-09-07

**Authors:** Maria F. Gottardo, Carla S. Capobianco, Johanna E. Sidabra, Juan Garona, Yasser Perera, Silvio E. Perea, Daniel F. Alonso, Hernan G. Farina

**Affiliations:** 1 Laboratory of Molecular Oncology, Department of Science and Technology, National University of Quilmes, Roque Saenz Peña 352, B1876BXD Bernal, Buenos Aires Argentina; 2 Laboratory of Molecular Oncology, Division of Pharmaceuticals, Center for Genetic Engineering and Biotechnology (CIGB), Havana, Cuba

**Keywords:** Breast cancer, Metastasis

## Abstract

CK2 is a serine/threonine kinase that is overexpressed in breast cancer and its inhibition is associated to reduced tumor growth and disease progression. CIGB-300 is an antitumor peptide with a novel mechanism of action, since it binds to protein kinase CK2 catalytic subunit alpha and to CK2 substrates thus preventing the enzyme activity. Our aim was to evaluate the potential therapeutic benefits of CIGB-300 on breast cancer disease using experimental models with translational relevance. We demonstrated that CIGB-300 reduces breast cancer cell growth in MDA-MB-231, MCF-7 and F3II cells, exerting a pro-apoptotic action and cell cycle arrest. We also found that CIGB-300 decreased cell adhesion, migration and clonogenic capacity of malignant cells. Effect on experimental breast cancer lung metastasis was evaluated after surgical removal of primary F3II tumors or after tail vein injection of tumor cells, also we evaluated CIGB-300 effect on spontaneous lung metastasis in an orthotopic model. Systemic CIGB-300 treatment inhibited breast cancer colonization of the lung, reducing the size and number of metastatic lesions. The present preclinical study establishes for the first time the efficacy of CIGB-300 on breast cancer. These encouraging results suggest that CIGB-300 could be used for the management of breast cancer as an adjuvant therapy after surgery, limiting tumor metastatic spread and thus protecting the patient from distant recurrence.

## Introduction

CK2 (Casein kinase 2) is a constitutive and pleiotropic enzyme whose function is the phosphorylation of serine and threonine residues in numerous proteins. With more than three hundred substrates, this kinase is involved in a large number of cellular functions, such as replication, cell proliferation, differentiation, regulation of apoptosis and angiogenesis^1–3^. CK2 is overexpressed in a wide variety of human neoplasms such as NSCLC, colorectal, prostate, head and neck, and breast cancer^2,4^, being mammary adenocarcinomas the tumor type with the highest values of CK2 overexpression (up to eighteen times overexpressed)^5^. High expression levels of CK2 in tumors correlate with increased angiogenesis and metastatic potential, undifferentiated histological type, multidrug-resistance phenotypes and poor clinical outcome^6–9^. In addition, CK2 overexpression has been reported as a possible cause of malignant transformation, giving the transformed cells the ability to evade apoptosis through the activation of survival signaling pathways^9–11^. Considering the oncogenic potential of CK2 enzyme and that the inhibition of its activity or expression has a detrimental effect on neoplastic cells^12^, numerous therapeutic strategies targeting CK2 have been developed.

The CIGB-300 peptide is a synthetic chimera originally developed by Perea in 2004, at the Center for Genetic Engineering and Biotechnology of Havana, Cuba. It contains a cyclic peptide that acts as a substrate inhibitor, targeting the phospho-acceptor domain of CK2 substrates, and thus preventing the correct phosphorylation by the enzyme^13,14^ and interacts with CK2 catalytic subunit alpha and blocks the enzymatic activity of kinase^15^. This inhibitor, which was obtained by using a peptide phage library against the CK2 phospho-acceptor site of the HPV-16 E7 oncoprotein, is fused to the Tat cell penetrating peptide. The biological effects and preclinical antitumor activity of CIGB-300 were previously explored in several types of cancer including SCLC, NSCLC and cervical cancer, among others^16–18^. CIGB-300 is a strong inducer of apoptosis in tumor cells and this proapoptotic activity is related to the nucleolar disassembly as a consequence of the inhibition of nucleophosmin/B23 phosphorylation^16–20^. It was recently published that this peptide is also capable of inhibiting proliferation, adhesion and invasion of lung cancer cells by affecting key signaling pathways associated with malignant progression^21,22^. Moreover, using a chemoresistance model of NSCLC this peptide showed improved effectiveness in comparison to cisplatin-sensitive cells associated with NF-κB inhibition^22^. As showed in several preclinical studies using cervical and lung cancer models, systemic administration of CIGB-300 in syngenic mice is associated to a marked reduction of tumor growth and angiogenesis, and also an inhibition of cancer cell dissemination and distant organ colonization^16,20,21^.

Breast cancer is one of the most prevalent cancers, and in turn, is the leading cause of cancer deaths in women around the world. Breast tumors present a heterogeneous biological behavior and a great variety of clinical features^23^. Considering that CIGB-300 has shown a strong anticancer activity through inhibition of CK2 in several experimental cancer settings and breast cancer is one of the main tumor types in which CK2 is overexpressed, the aim of the present study was to test the preclinical efficacy of CIGB-30 using clinically relevant breast cancer models. For this purpose, we evaluated the effect of CIGB-300 on several key aspects of cancer cell biology emphasizing metastatic progression, using three breast cancer models with different molecular profiles and aggressiveness phenotypes. Our results showed that CIGB-300 strongly inhibits breast cancer cell growth and impairs in vitro colony formation in all evaluated cell lines. Direct cytostatic effect of CIGB-300 was associated to a strong induction of apoptosis and cell cycle arrest in S or G_0_–G_1_ phase, depending on tumor cell type. Additionally, short-term exposure to the peptide reduces breast cancer cell adhesion and migration in comparison to vehicle-treated cultures. In vivo, CIGB-300 systemic treatment was capable of reducing cancer cell dissemination and pulmonary colonization in different experimental and spontaneous models of metastatic spread. Interestingly, antimetastatic efficacy of the peptide was greater after evaluating the establishment and growth of postsurgical macrometastatic lung tumors. Taking altogether, the evidence presented in this work shows a strong therapeutic potential of CIGB-300 supporting further development and evaluation of the peptide as an antitumor agent for breast cancer.

## Results

### CIGB-300 reduces cell growth in breast cancer cells

**Figure 1:**
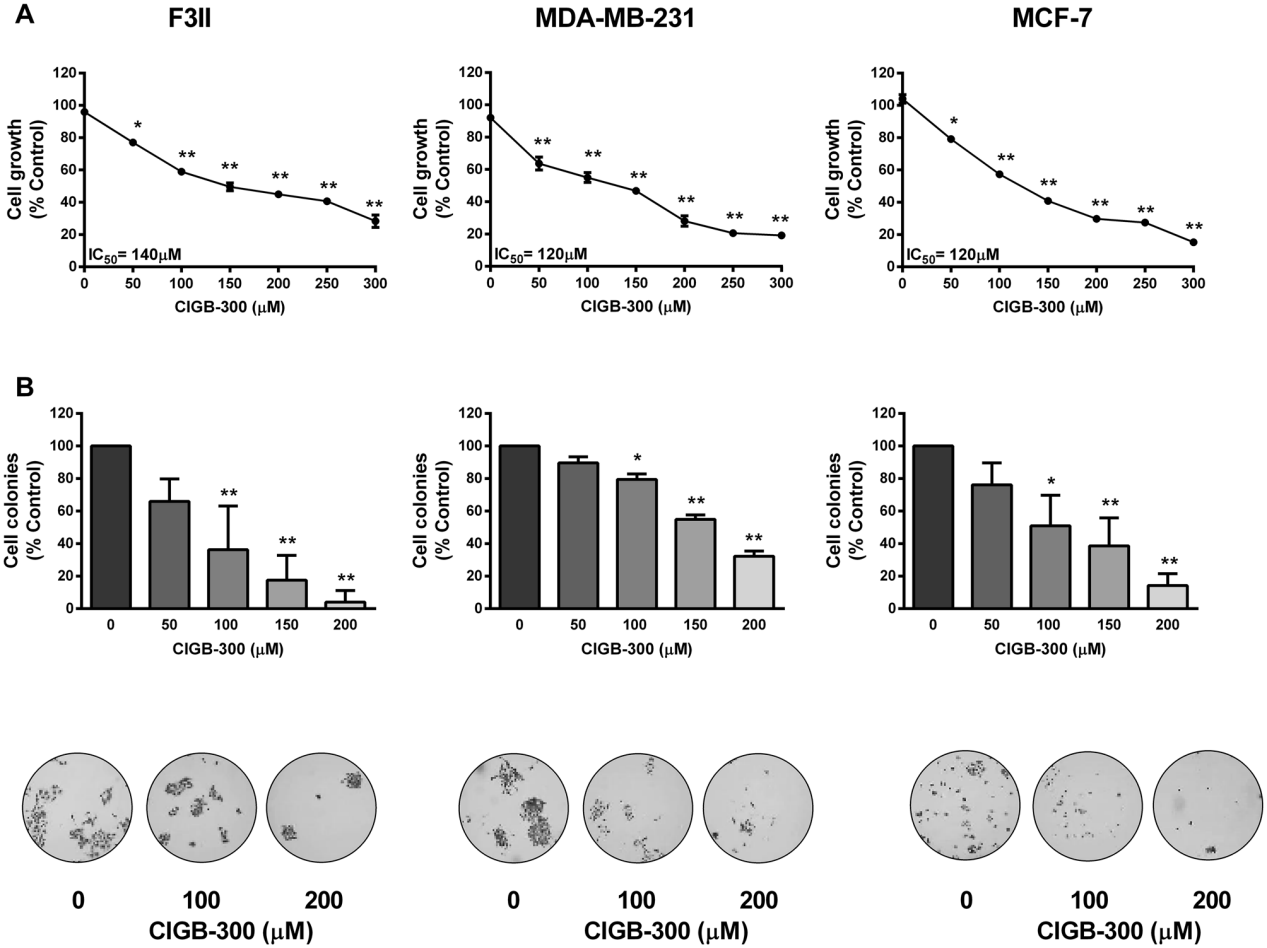
CIGB-300 inhibits cell growth in breast cancer cells. (**A**) F3II, MDA-MB-231 and MCF-7 cells were treated with increasing concentrations of CIGB-300 ranging from 0 to 150 μM o for 72 h. Cell growth was determined by crystal violet colorimetric method. Each point represents the average of six independent measurements, each done in triplicate with the SEM. (**B**) Quantitative analyses of number of colonies per quadrant are also shown. Representative microphotographs of the effect of 7-day treatment of CIGB-300 on the colony formation assay at low density (bottom). Values represent mean ± SEM. Results are representative of three independent experiments *p < 0.05; **p < 0.01 versus control. ANOVA, Dunnett’s Multiple comparison test.

Since CK2 is overexpressed in breast cancer and previous reports describe the antiproliferative and proapoptotic effects of CIGB-300 on several cancer cell lines, we first evaluated the effect of CIGB-300 on breast cancer cellular growth (Fig. 1A). We first observed that CIGB-300 caused a marked and dose-dependent reduction of cell growth in all tested breast cancer cell lines after 72 h incubation. The obtained IC_50_ values were 140 μM for F3II, and 120 μM for both, MDA-MB-231 and MCF-7, showing an equivalent cytostatic potency in all evaluated cell models. These values were taken into account to define CIGB-300 concentrations assayed in further in vitro protocols as approximately ½, 1 and twofold increase of IC_50_ concentrations.

Growth-modulating activity was also evaluated in low density breast cancer cell cultures exposed to CIGB-300 during 1 week (Fig. 1B). Long-term in vitro treatment using CIGB-300 was able to drastically impair breast cancer colony formation reducing up to ~ 96, ~ 68 and ~ 88% the clonogenic growth in F3II, MDA-MB-231 and MCF-7 cells, respectively. In addition, lack of unspecific cytotoxic effects of the peptide after 24 h was confirmed using concentrations between 0 and 300 μM (Supplemental Fig. 1).

### CIGB-300 increases breast cancer cell apoptosis

**Figure 2:**
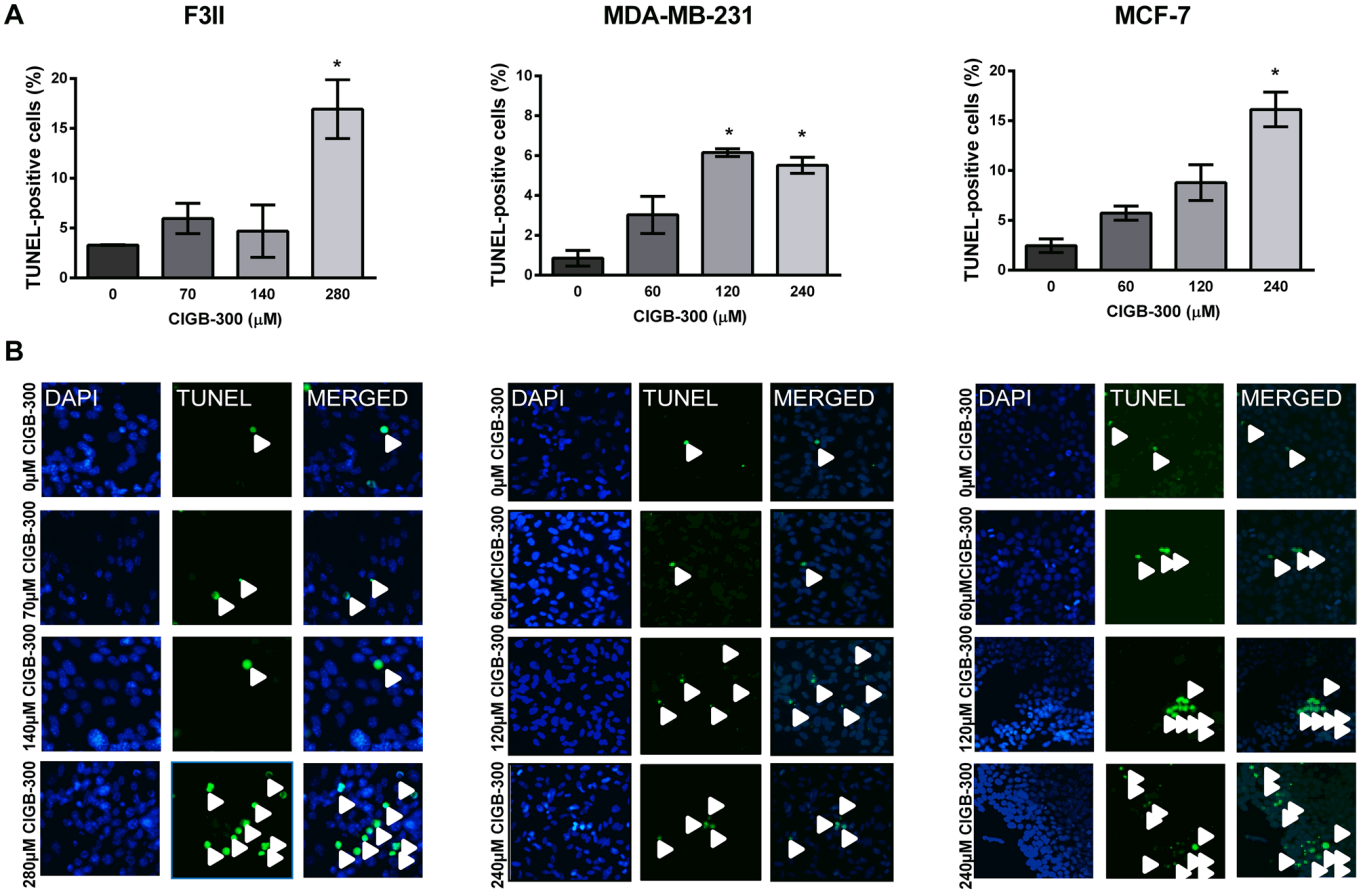
CIGB-300 exerts a proapoptotic action on breast cancer cells. (**A**) F3II, MDA-MB-231 and MCF-7 cells were incubated with CIGB-300 for 24 h. Apoptosis was assessed by TUNEL method. Each column represents the percentage ± CL of TUNEL-positive cells (n ≥ 1,000 cells/group). Data from three separate experiments were analyzed by χ^2^ test. *p < 0.05 versus control. (**B**) Representative photographs of F3II, MDA-MB-231 and MCF-7 cells after the treatment with CIGB-300 (original magnification × 100).

As above-mentioned, it is known that CK2 has more than 300 substrates implicated in a large number of cellular functions both in normal and transformed tissues, such us replication, proliferation, differentiation and the regulation of programmed cell death. Moreover, it has been previously reported that CIGB-300 is capable of triggering apoptosis in malignant lung cells as well as in vascular endothelial cells^16,20^. Consequently, we further explored whether CIGB-300 peptide may activate the apoptotic process in breast cancer cells. For that purpose we used the TUNEL assay, which detects DNA breakage that occurs during late stage of apoptosis. As observed in Fig. 2, CIGB-300 triggers apoptosis after 24 h incubation, presenting a significant proapoptotic effect starting at 280, 120 and 240 µM in F3II, MDA-MB-231 and MCF-7 cells, respectively (Fig. 2A–C).

### CIGB-300 arrests cell cycle in breast cancer cells

**Figure 3:**
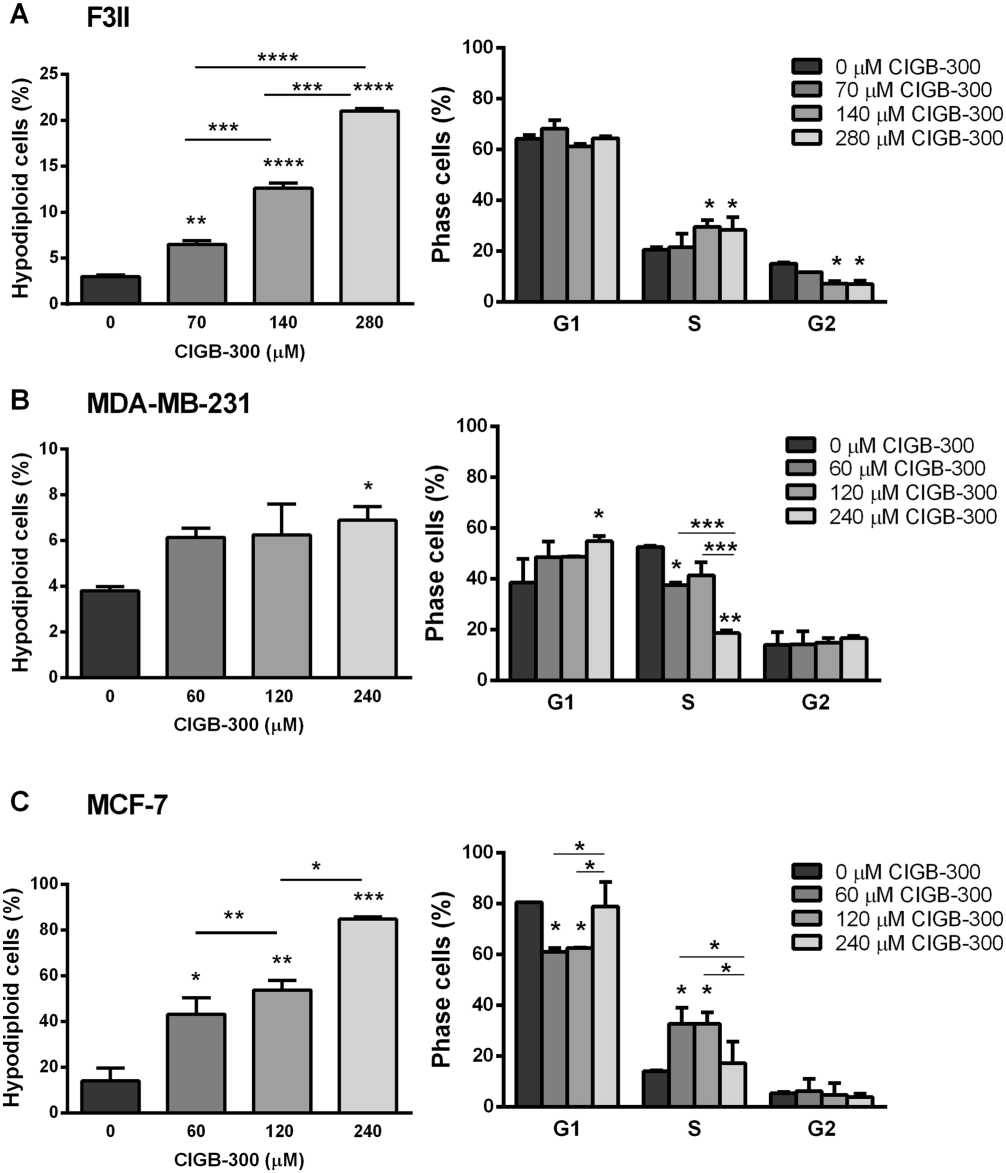
Effect of CIGB-300 on cell cycle. Cell cycle distribution of (**A**) F3II, (**B**) MDA-MB-231 or (**C**) MCF-7 cells was analyzed by FACS using PI (right) Additionally, cell apoptosis was determined as the percentage of hypodiploid cells (left). Each column represents the mean ± SEM of the percentage of sub G_0_–G_1_–, G_0_/G_1_–, S– and G2/M-phase cells. *p < 0.05; **p < 0.01 versus control. ANOVA, Tukey’s multiple comparisons test.

CK2 has been widely characterized as an important regulator of the cell cycle. After analysis of cell cycle distribution using flow cytometry, we observed that CIGB-300 treatment resulted in an increased of sub-G_0_ phase in all evaluated cell lines, as determined by the percentage of cells with hypodiploid DNA content (Fig. 3A–C, left). This result is in line with above-described proapoptotic activity of the peptide given that sub-G_0_ population of cells is often used to estimate apoptosis on the basis of their reduced DNA content. Interestingly, treatment with CIGB-300 induced a significant S phase cell cycle arrest in the F3II and MCF-7 cell lines, whereas in MDA-MB-231 cells peptide caused a significant arrest in G_0_/G_1_ phase, indicating that CIGB-300 effect on cell cycle distribution is cancer cell type dependent (Fig. 3A–C, right).

### CIGB-300 inhibits the migratory capability and cell adhesion in breast cancer cells

**Figure 4:**
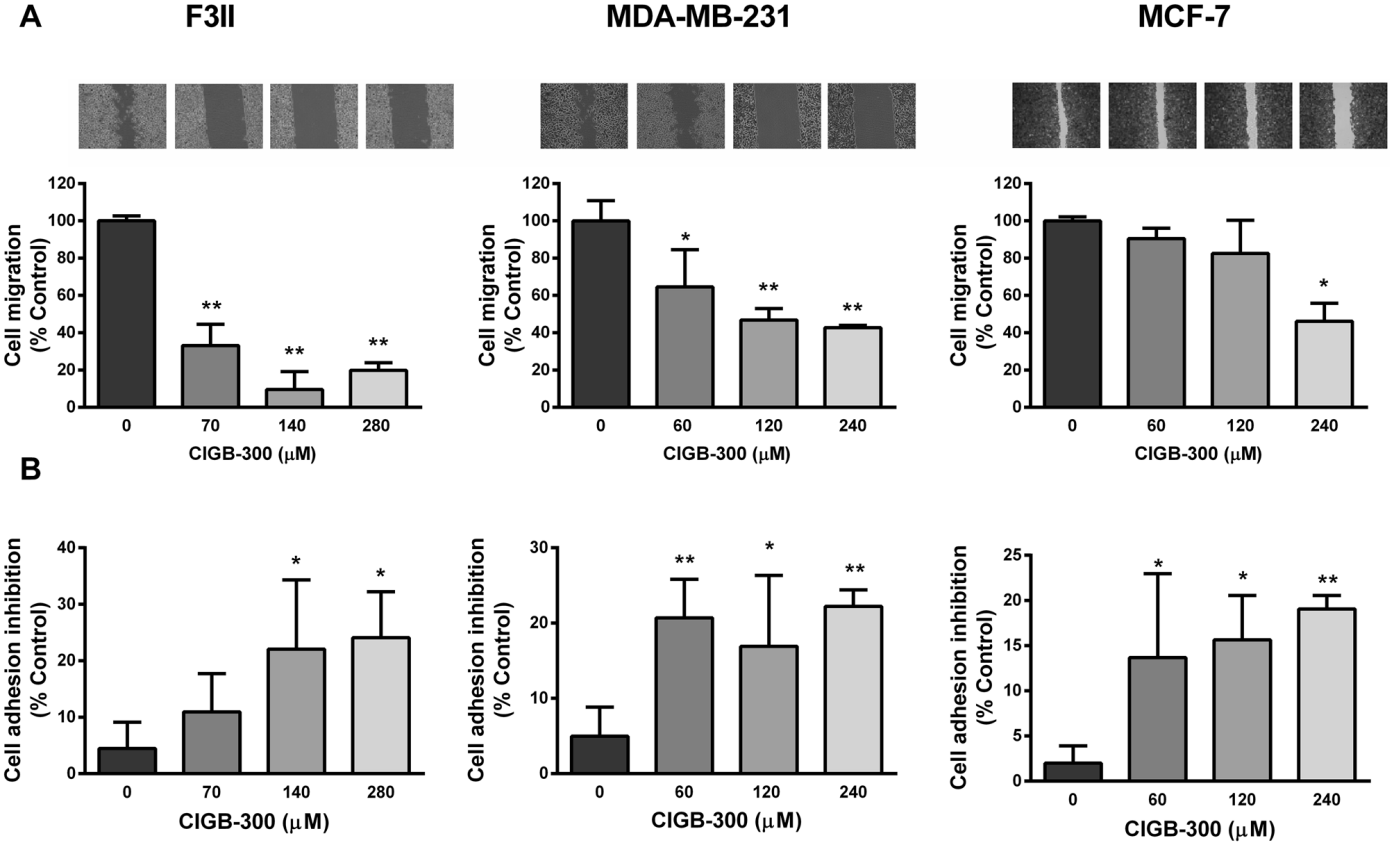
CIGB-300 treatment impairs breast cancer cell migration and adhesion. (**A**) Confluent monolayers of F3II, MDA-MB-231 and MCF-7 cells were wounded and CIGB-300 was added at different concentrations during 20 h. Wound closure was measured in five photographed representative fields, and control group was taken as 100%. Data are presented as mean ± SD, and are representative of three independent experiments. (**B**) For cell adhesion: cells were seeded and incubated with CIGB-300 for 2 h at 37 °C. Cell adhesion was determined by a colorimetric method. Data represent the means ± SEM of at least three independent experiments and were expressed as percentage of adhesion respect to the control. *p < 0.05; **p < 0.01 versus control. ANOVA, Dunnett’s Multiple comparison test.

Cell migration and adhesion are key processes in many relevant pathological scenarios and are fundamental aspects of the metastatic process. As shown in Fig. 4A, CIGB-300 produced a significant inhibition of migratory capacity of breast cancer cells. In MDA-MB-231 and F3II cell lines, CIGB-300 at the referenced IC_50_ concentrations reduced migration by 50 and 90%, respectively. In the less aggressive and invasive MCF-7 cell line, antimigratory activity of the peptide was less potent reaching statistical significance only at 240 μM. Furthermore, we evaluated the effect of CIGB-300 on cancer cell adhesion. Treatment with 240 or 280 μM concentrations of CIGB-300 was able to reduce up to ~ 20% breast cancer cell adherence after a 2 h exposure to the peptide (Fig. 4B).

### CIGB-300 systemic treatment inhibits breast cancer lung metastasis

**Figure 5:**
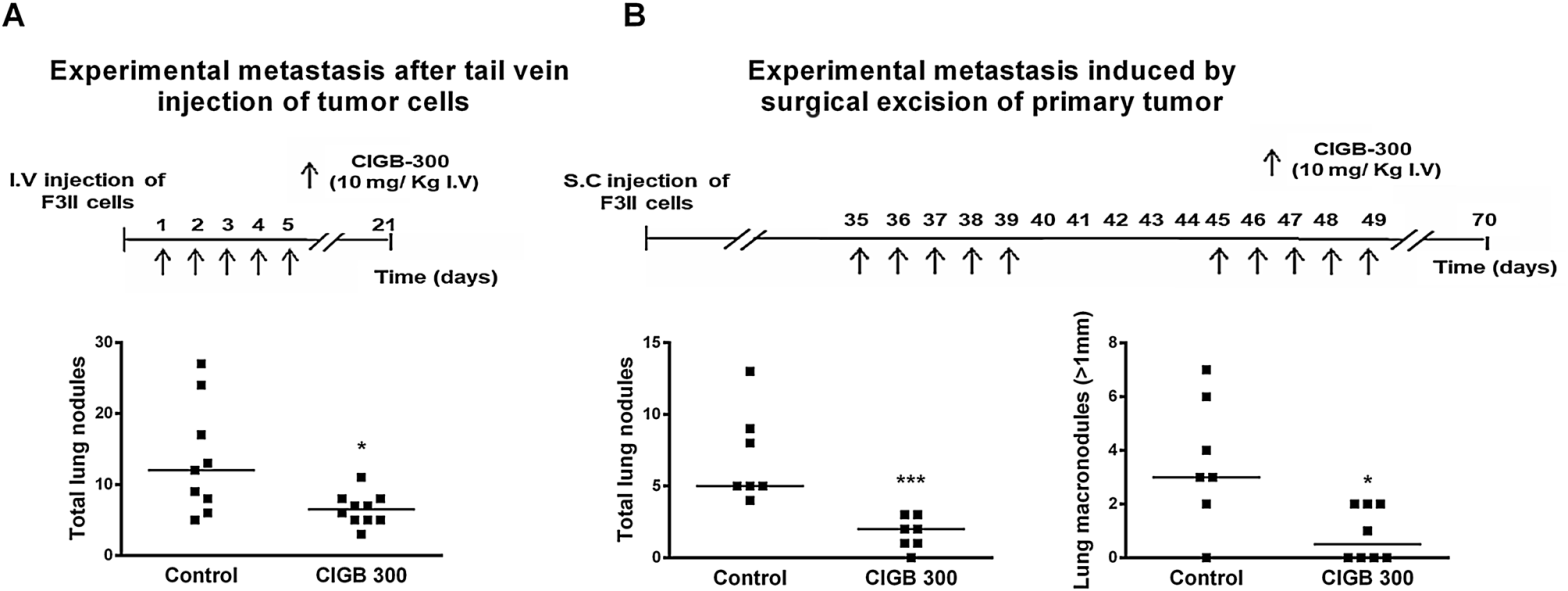
CIGB-300 treatment inhibits breast cancer metastasis to lung. (**A**) *Tail vein experimental metastasis:* F3II cells were injected in the lateral tail vein of mice. Mice were treated with CIGB-300 at a 10 mg/kg i.v. dose, during five consecutive days, and sacrificed at day 21. The number of lung nodules per animal is represented in the graph. Data are expressed as scatter dot plot and median (n = 9–10 per experimental group). **p < 0.01, Unpaired Mann–Whitney test. (**B**)* Experimental metastasis after incomplete surgical resection of primary tumor*: F3II cells were injected subcutaneously in the right flank of BALB/c mice to generate primary tumors. On day 35 after tumor cell inoculation, an incomplete surgical resection of the tumor was performed. Immediately after surgery mice were treated daily with 10 mg/kg i.v. dose of CIGB-300 five consecutive days after surgery, and five consecutive days after day 10 post-surgery. On day 70 animals were sacrificed. n = 7–8 per experimental group. *p < 0.05 and ***p < 0.001, **p < 0.01, Unpaired Mann–Whitney test.

Metastasis is the primary cause of breast cancer-related deaths worldwide. In addition, lung metastasis is one of the most common distant metastases of breast cancer, and is strongly associated to poor prognosis^24^. In order to assess the effect of CIGB-300 on breast cancer metastatic spread to lung we first evaluated its impact on pulmonary experimental metastasis after injecting F3II cells in the lateral tail vein of syngenic immunocompetent BALB/c mice. During five consecutive days, mice were treated with CIGB-300 at a concentration of 10 mg/kg i.v. and after 21 days of cancer cell injection mice were sacrificed and lung nodules were counted. Interestingly, CIGB-300 treatment significantly inhibited the development of pulmonary nodules compared to control group, reducing nearly ~ 45% the number of metastatic lesions (Fig. 5A).

**Figure 6:**
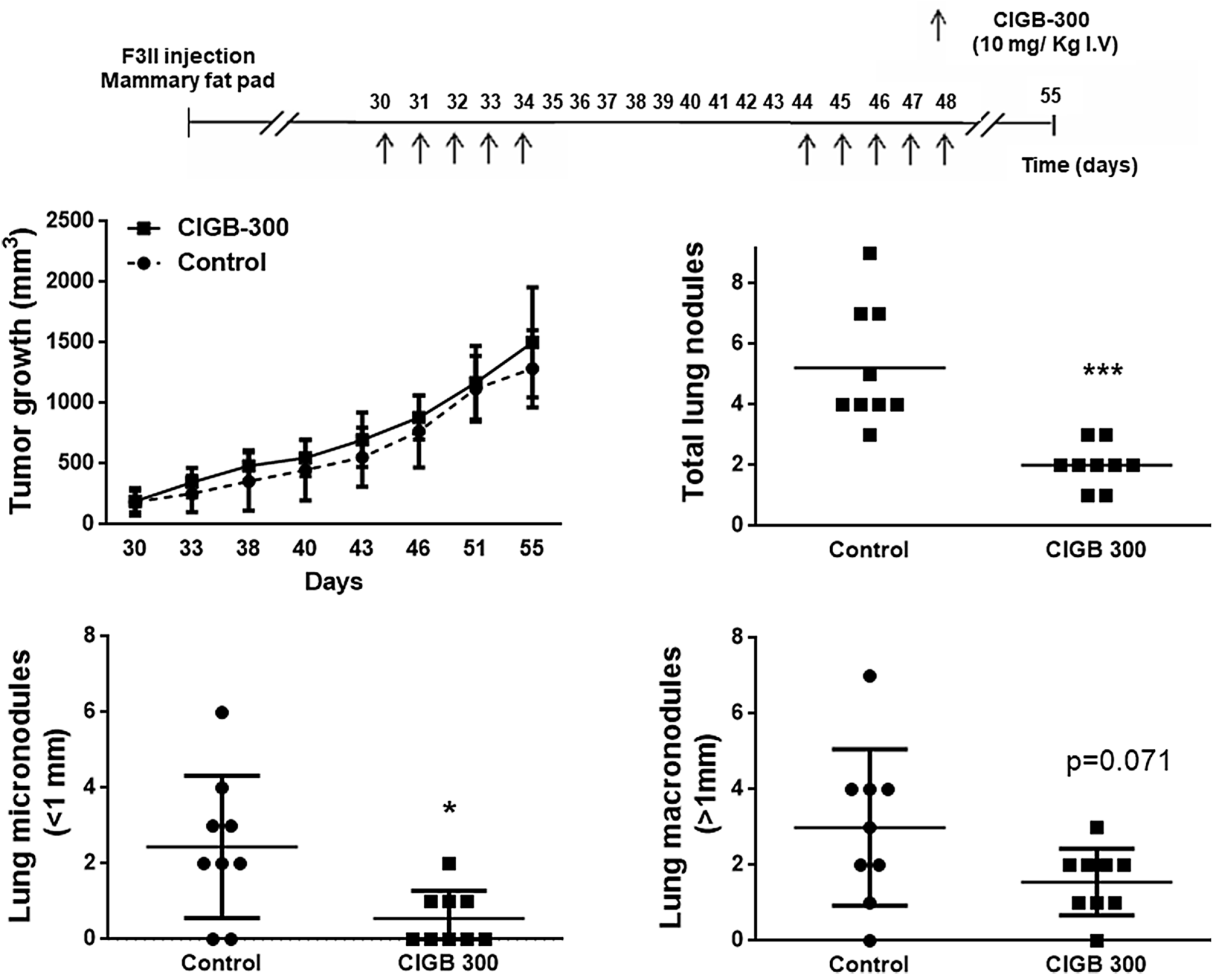
CIGB-300 treatment inhibits spontaneous breast cancer lung metastasis. CIGB-300 treatment inhibits spontaneous lung metastasis in a breast cancer orthotopic model. F3II cells were injected into the mammary fat pad of syngeneic BALB/c mice to generate primary tumors. On day 30 after tumor cell inoculation, mice were treated with a 10 mg/kg i.v. dose of CIGB-300 for five consecutive days. A second round of treatment was conducted 44 days after inoculation. On day 55 animals were sacrificed. n = 9 per experimental group. *p < 0.05 and ***p < 0.001, Unpaired Mann–Whitney test.

Secondly, we determined whether treatment with CIGB-300 could interfere in the development of local recurrences and lung metastatic lesions after incomplete surgical removal of F3II mammary tumors. In order to evaluate the adjuvant anticancer efficacy of the peptide, syngeneic F3II primary tumors were generated and resected on reaching a predetermined size, and CIGB-300 was administered in two cycles of five 10 mg/kg i.v. daily doses after tumor resection. As shown in Fig. 5B (left), adjuvant systemic treatment using i.v. CIGB-300 severely impaired postoperative pulmonary metastasis, causing a ~ 60% reduction in the total number of lung nodules. No difference in local recurrence growth was observed between experimental groups (Data not shown). Antimetastatic activity of CIGB-300 was greater when macrometastatic disease was assessed (metastatic nodules larger than 1 mm in diameter), inhibiting by 83% the number of secondary F3II lung lesions in comparison to vehicle-treated animals (Fig. 5B, right). CIGB-300 treatment was also associated to a reduction in the prevalence of mice bearing macrometastatic disease (85.7% versus 42.8%, control versus CIGB-300, respectively) and mice with heavily colonized lungs (71.4% vs 0%, control vs CIGB-300, respectively). Finally, to confirm the inhibitory effect of CIGB-300 treatment on breast cancer metastatic growth, we conducted an orthotopic inoculation of F3II cells. Our results demonstrated that CIGB-300 treatment significantly inhibited the development of pulmonary nodules compared to control group, reducing nearly ~ 40% the number of total metastatic lesions. Also, we demonstrate that CIGB-300 significantly reduced micrometastasis (metastatic nodules lower than 1 mm in diameter). Furthermore, after CIGB-300 treatment only 45% of mice developed micrometastasis versus 80% of mice bearing micrometastasis for the control group. Although not in a statistically significant manner, the treatment with CIGB-300 decreased the number of macro-metastasis compared to control group (*p* value = 0.071). As we expected, no difference in tumor growth was observed between experimental groups (Fig. 6).

## Discussion

Protein kinase CK2, is a serine–threonine kinase frequently upregulated and overexpressed in several types of human tumors. CIGB-300 is a peptidic inhibitor of CK2, designed to bind to the phospho-acceptor domain of CK2 substrates, impairing the correct phosphorylation by the enzyme. Because breast cancer is one of the main tumor types in which CK2 is overexpressed, and CIGB-300 exerts an anti-metastatic effect in lung and cervix cancer, our aim was to evaluate the potential therapeutic benefits of CIGB-300 on breast cancer using experimental models with translational relevance.

Breast cancer is the most common malignant disease in women worldwide^25^. New strategies have been developed for the treatment of breast tumors that are positive for ER, PR and/or overexpress HER2/neu. However, a considerable number of patients will relapse as a result of metastasis, especially those with TNBC which has the worst prognosis. In particular, 60–70% of metastatic breast cancer patients who eventually die are diagnosed with lung metastasis, being the incidence of TNBC up to 40%^26–29^. Thus, it is necessary to develop new therapeutic strategies to improve the treatment of TNBC. For that purpose, we evaluated CIGB-300 effect in breast cancer cells using two aggressive TNBC models; the human MDA-MB-231 and the murine and highly metastatic F3II cell line. Additionally, MCF-7 cells were used as a hormone-responsive and much less aggressive experimental model.

It has been reported that CIGB-300 has a strong antitumor effect in lung and cervix cancers by reducing tumor growth, angiogenesis and spread to distant organs^18,19,21^. In this work, we evaluated the therapeutic efficacy of CIGB-300 treatment on breast cancer cell dissemination and lung colonization in vivo with two experimental metastatic models. We first evaluated the antimetastatic effect of CIGB-300 on TNBC cells using the tail vein metastasis assay. In this experimental scenario we showed that CIGB-300 treatment was associated to a significant reduction in the number of tumor nodules in the lungs of syngenic mice showing that CK2 may play a key role in the process of breast cancer cell implantation and outgrowth in pulmonary tissue. TNBC is typically treated with a combination of surgery, radiation therapy, and chemotherapy. Despite surgery is commonly the first step toward long-term control of breast cancer, recent evidence demonstrated that perioperative factors such as tumor cell dissemination, inflammation and release of proangiogenic factors may favor a permissive environment for the regrowth of local or distant residual tumors^30–32^. Taking this into account, we evaluated whether CIGB-300 treatment during the early postoperative period can potentially minimize metastatic spread or survival of residual malignant cells after incomplete surgical removal of TNBC primary tumors. After 10 days of intravenous treatment using CIGB-300 pulmonary metastatic colonization by F3II cells was reduced by 60% in comparison to vehicle-treated mice. Furthermore, adjuvant CIGB-300 significantly impaired macrometastatic disease, reducing by half the prevalence of mice bearing large lung metastatic lesions. To conclude in vivo experiments, the therapeutic efficacy of CIGB-300 treatment in an orthotopic model was studied. It is widely known that this kind of tumor cell inoculation mimics with greater fidelity breast cancer disease. In this case, CIGB-300 was able to decrease breast cancer cell dissemination and metastasis. Of importance, this is the first time that an anti-CK2 targeted therapy shows efficacy in an orthotopic model of breast cancer.

In this study we also demonstrated that in vitro treatment with CIGB-300 significantly inhibited cell adhesion and migration in a dose-dependent manner in breast cancer cells. Both cellular phenomena are crucial events in tumor cell dissemination and metastatic outgrowth where CK2 plays a key role^13,22,33^. It has been reported that in head and neck squamous cell carcinoma CK2 modulates the expression of certain integrin genes involved in adhesion and migration^16^. Moreover, CIGB-300 has been reported to decrease cell adhesion and migration in both vascular endothelial HUVEC and lung cancer cells^18,21^. Tumor cell motility impairment by CIGB-300 correlated with the inhibition of the proteolytic activity of cancer cell-secreted uPA and MMPs, key molecular players in tumor invasion, angiogenesis and metastatic spread^18,21^.

In addition, we explored the direct antiproliferative effect of CIGB-300 on high and low density breast cancer cultures. CIGB-300 concentrations of 100 μM or higher were able to drastically inhibit tumor cell growth and colony forming ability. These findings are in agreement with previous studies in which CIGB-300 displayed cytostatic activity on a large number of tumor types including NSCLC, SCLC, epithelioma, hepatocellular carcinoma and cervical cancer amog others^16–18, 22^. One of the main mechanisms by which CK2 favors tumor development is apoptosis evation, through the activation of survival signaling pathways, and cell cycle progression. Different CK2 inhibitors that target the ATP-binding site on the catalytic subunit have previously demonstrated propapoptotic activity on malignant cells^13^. Here we demonstrated that CIGB-300 triggers apoptosis in all tested breast cancer cell models. Previous studies on the molecular and cellular events leading to apoptosis in CIGB-300-treated cancer cells showed that CIGB-300 is transported from the cytoplasm to the nucleus and when the CIGB-300 reaches the nucleolus, it inhibits the CK2-mediated phosphorylation of the multifunctional nucleolar protein B23/nucleophosmin (NPM) inducing a nucleolar disassembly leading to apoptosis^10,17^. It was also shown that pharmacologic inhibition of such phosphorylation event by the peptide modulates genes related to protein synthesis, mitochondrial ATP metabolism, and ribosome biogenesis^18^. However, the specific mechanisms responsible for the proapototic activity of the peptide in breast cancer remain to be elucidated.

In addition to its ability to increase programmed cell death and impair tumor cell proliferation, CIGB-300 treatment of breast cancer cells was also associated to cell cycle arrest in the S- or G_0_/G_1_-phase, showing that specific regulation of cell cycle distribution is cell line dependent. Despite the fact that direct cytostatic action of CIGB-300 on malignant cells was previously reported to be specifically mediated by CK2 substrate- and enzyme-binding mechanism^15–17^, we cannot assure that other *off* target effects of the peptide are not in part responsible for the anti-breast cancer activity described in the present study. In this regard and with the aim of assessing the specific involvement of the targeted kinase in breast cancer, studies showing down-regulation of CK2 activity after CIGB-300 treatment, for example via phosphorylation inhibition of confirmed substrates, are mandatory and will be performed in the near future.

All in vitro and in vivo results indicated that treatment with CIGB-300 inhibits crucial cellular and molecular events related to breast cancer progression and metastatic spread, and these multiple anticancer effects may be collectively involved in the robust antimetastatic activity of CIGB-300. Considering its multiple anticancer effects, this peptide may be used as an adjuvant therapy after surgery, limiting tumor metastatic spread and thus protecting the patient from distant recurrence.

## Methods

### Peptide synthesis

The peptide CIGB-300 used in this work was synthesized as previously described^14^.

### Breast cancer cell lines

Human breast carcinoma cell lines MDA-MB-231 (ATCC HTB-26) and MCF-7 (ATCC HTB-22) were obtained from the American Type Culture Collection. MDA-MB-231 is a triple-negative breast cancer (TNBC) cell line which lacks the estrogen receptor (ER) and progesterone receptor (PR), and expresses low levels of human epidermal growth factor receptor 2 (HER2)/neu. It also belongs to the claudin-low molecular subtype. MCF-7 is a ER-positive/ PR-positive luminal mammary carcinoma^34^. CK2 is overexpressed in breast cancer cell lines, including MCF-7 and MD-MB-231, which were used in the present work, has been reported in previous studies^35^. The F3II mammary carcinoma cell line is a highly invasive and metastatic variant derived from a clone of a spontaneous BALB/c mouse mammary tumor^36^. It is a hormone-independent tumor cell line and expresses low levels of HER2/neu. Tumor cells were grown in Dulbecco's modified Eagle’s medium (DMEM, Gibco, Rockville, MD, USA) plus 10% fetal bovine serum (FBS) and 40 mg/ml gentamycin (Fada Pharma, Argentina) in monolayer culture, at 37 °C in a humidified atmosphere of 5% CO2. All cells were harvested using a tripsin/EDTA solution (Gibco) diluted in phosphate-buffered saline (PBS).

### Tumor cell growth

Cells were seeded into 96-well plates at a density of 2 × 10^3^ for F3II and 2.5 × 10^3^ for MDA-MB-231 and MCF-7 cells per well and incubated for 24 h. After 24 h, CIGB-300 concentrations ranging from 0 to 150 μM in complete medium were added to the wells. After incubation for 72 h, medium was removed and the plates were washed with PBS. Then, attached cells were fixed in methanol for 15 min and stained with a 0.5% crystal violet solution for another 15 min. To remove excess dye, cells were washed with rinse water. The dye in the stained cells was dissolved in 10% methanol—5% acetic acid solution (v/v) and absorbance at 595 nm was measured in a 96-well plate reader (ASYS Hitech GmbH, Austria). The concentration producing 50% inhibition (IC_50_) was determined by non-linear regression function of GraphPad Prism6®. Results shown correspond to the average of three separate experiments, including six experimental replicates for each condition, and SEM was determined. The optical density of vehicle-treated control cells was taken as 100%.

Lack of direct cytotoxicity of CIGB-300 at shorter periods of incubation was confirmed before evaluating its effects on apoptosis, cell cycle and migration. In contrast with the above mentioned protocol, for cytotoxicity determinations, tumor cells were seeded into 96-well plates at a density of 4 × 10^3^ for F3II or 5 × 10^3^ for MDA-MB-231 and MCF-7 cells per well and incubated with CIGB-300 for 24 h.

Cytostatic effects of CIGB-300 were studied at low cell density by colony formation assay, as we previously described^37^. Cells were plated at 6 × 10^2^ cells/well in 24-well plates and grown for 7 days in complete medium with CIGB-300. Cultures were then stained with crystal violet and colonies of more than 50 tumor cells were counted.

### Apoptosis

F3II, MDA-MB-231 and MCF-7 cells were cultured on glass cover slips, and incubated with CIGB-300 (70, 140 and 280 μM for F3II and 60, 120, 240 μM for MDA-MB231 and MCF-7) for 24 h, and then stained according to manufacturer’s protocol. Apoptotic cells were detected by terminal deoxynucleotidyl transferase dUTP nick end labeling (TUNEL) using the DeadEnd Fluorometric TUNEL System (Promega Corporation, Fitchburg, WI, USA).The slides were mounted with Vectashield (Vector Laboratories, Burlingame, CA) containing 4,6-diamidino-2-phenylindoledihydrochloride (DAPI) for DNA staining and visualized in a fluorescent light microscope (Cytation). The percentage of apoptotic cells was calculated as [(TUNEL +)/total cells] × 100. Apoptotic cells were detected using the TUNEL method as we previously described^38^.

### DNA-cell cycle analysis

Cell cycle phase distribution was evaluated by flow cytometry as we previously described^39^. F3II, MDA-MB-231 and MCF-7 cells were cultured with CIGB-300 (70, 140 and 280 μM for F3II and 60, 120, 240 μM for MDA-MB231 and MCF-7) for 24 h and were fixed in ice-cold 70% ethanol. DNA was stained with propidium iodide (PI, 50 µg/ml) in PBS containing ribonuclease (10 µg/ml) for 20 min at 37 °C. Cell cycle phase distribution of nuclear DNA was carried out in a FACsCalibur cytometer using WinMDI 2.9 and Cylcherd 1.2 softwares. Cells with PI staining intensity lower than the G0/G1 peak were considered hypodiploid.

### Wound migration assay

Cell migration was assessed as previously described^21^. F3II, MDA-MB-231 and MCF-7 cells were seeded at a density of 1 × 10^6^ cells/well in a 6-well plate. When cells reached 95% confluence, wounds were made in the cell monolayer. Plates were incubated during 20 h in presence of CIGB-300 (70, 140, 280 µM for F3II cells, or 60, 120, 240 µM for MDA-MB-231 and MCF-7). 1 h prior to assay finalization, control wounds were made. After that, cells were washed with PBS, fixed with 10% formalin and stained with crystal violet dye (0.5%). Photographs were taken using a camera connected to the Nikon TE-2000 inverted microscope (Nikon, NIS elements software), and wound closure area was quantified using Image J software. The wounded area covered by vehicle-treated control cells was taken as 100%.

### Adhesion assay

Cell adhesion was assessed as previously described^21^. Cells were seeded at a density of 2 × 10^4^ cells/well or 2.5 × 10^4^ cells/well for F3II or MDA-MB-231/MCF-7 respectively in 96-well plates. After cells were treated with CIGB-300 (F3II: 70, 140, 280 µM and MDA-MB 231 and MCF-7: 60, 120, 240 µM) for 2 h, medium was removed and the plates were washed with PBS. Then, attached cells were fixed with methanol for 15 min and stained with a crystal violet solution 0.5% for another 15 min. The excess dye was removed by washing with rinse water. The dye in the cells was dissolved in 10% methanol—5% acetic acid solution (v/v) and the absorbance was measured at 595 nm in a 96-well plate reader (ASYS Hitech GmbH, Austria). Each condition was assayed in sextuplicate in two different experiments, and SEM was determined. The optical density corresponding to vehicle-treated control attached cells was taken as 100%.

### Animals

Eight weeks old BALB/c mice were purchased from Centro de Medicina Comparada (ICiVet-CONICET UNL, Argentina), and kept 5–8 mice per cage in the animal house facility at the National University of Quilmes. Food and water was provided ad libitum and general health status of the animals was monitored daily. All animal procedures were conducted according to the NIH guidelines and regulations and approved by the Institutional Animal Care Committee, National University of Quilmes.

### Experimental metastasis following tail vein injection of breast cancer cells

To study CIGB-300 effect on experimental F3II lung colonization, 2 × 10^5^ cells were injected into the lateral tail vein of BALB/c mice as previously described^40^. During the next 5 days, mice were treated intravenously with 10 mg/kg CIGB-300 in PBS. Control animals were injected i.v. with the vehicle (PBS). On day 21, lungs were excised and fixed in Bouin’s solution and metastatic lung nodules were counted under a dissecting microscope.

### Experimental metastasis after incomplete surgical resection of primary tumor

With the aim of studying CIGB-300 effect on the development of postsurgical local recurrences and metastatic disease, F3II tumors were generated after injecting 2 × 10^5^ cells in the subcutis of the right flank of syngenic BALB/c mice as previously described^39^. At day 35, an incomplete resection of primary tumors was performed, leaving a standardized tumor piece (1 × 1 mm^3^) in the subcutis. Mice were treated daily with 10 mg/kg i.v. dose of CIGB-300 five consecutive days after surgery, and five consecutive days from day 10 post-surgery. Control animals received only saline vehicle. After 35 days from surgery, animals were sacrificed by cervical dislocation, lungs were excised and fixed in Bouin’s solution and lung nodules were counted under a dissecting microscope. Lung metastatic lesions were classified according to their diameter in small nodules when < 1 mm or in large macrometastatic nodules when > 1 mm. Prevalence (%) of mice bearing macrometastatic disease (positive for nodules > 1 mm in diameter) and mice with heavily colonized lungs (positive for more than 2 macrometastatic nodules) was also calculated.

### Spontaneous lung metastasis in an orthotopic breast cancer model

To study CIGB-300 effect in an orthotopic model of breast cancer, 1 × 10^5^ F3II cells were inoculated into the mammary fat pad of BALB/c mice to obtain a solid tumor growth. Thirty days after inoculation, once the tumor size reached 200 mm^3^, mice were treated daily with 10 mg/kg i.v. dose of CIGB-300 for five consecutive days and at day 44 post-inoculation, mice received a second round of 10 mg/kg i.v. dose of CIGB-300 for five consecutive days. Tumor sizes were measured every 3 days. After 55 days post-inoculation, animals were sacrificed by cervical dislocation, lungs were excised and fixed in Bouin’s solution and lung nodules were counted under a dissecting microscope.

### Statistical analysis

PRISM 6, Version 6.01 (GraphPad software Inc., La Jolla, CA, USA) was used to conduct all statistical analyses. Tukey’s multiple comparisons test was used after ANOVA analysis (after confirming normal distribution of data) for the analysis between more than 2 experimental groups. The number of apoptotic cells evaluated by TUNEL in slides from three independent experiments was expressed as percentage of TUNEL positive cells ± 95% confidence limits (CL) of the total number of cells counted in each specific condition, and analyzed by χ^2^ test. Statistical analyses between 2 experimental groups were conducted using unpaired *t* test (weight of tumor recurrences in primary sites) or Mann–Whitney test (number of metastatic nodules). Differences were considered statistically significant at a level of *p* < 0.05. Data were presented as mean ± SEM or median (range).

## Supplementary information

Supplementary Figure 1.
