# Supplementary material for: Preclinical efficacy of CIGB-300, an anti-CK2 peptide, on breast cancer metastasic colonization
Source: Sci Rep. 2020 Sep 7;10:14689. doi: 10.1038/s41598-020-71854-6 (PMC7477577; doi:10.1038/s41598-020-71854-6)
Supplement: Supplementary file 1 — Supplementary Figure 1. [file 41598_2020_71854_MOESM1_ESM.pdf]

PRECLINICAL EFFICACY OF CIGB-300, AN ANTI-CK2 PEPTIDE, ON BREAST CANCER METASTASIC COLONIZATION

Maria F. Gottardo*, Carla S. Capobianco, Johanna E. Sidabra, Juan Garona, Yasser Perera, Silvio Perea, Daniel F. Alonso, Hernan G. Farina.

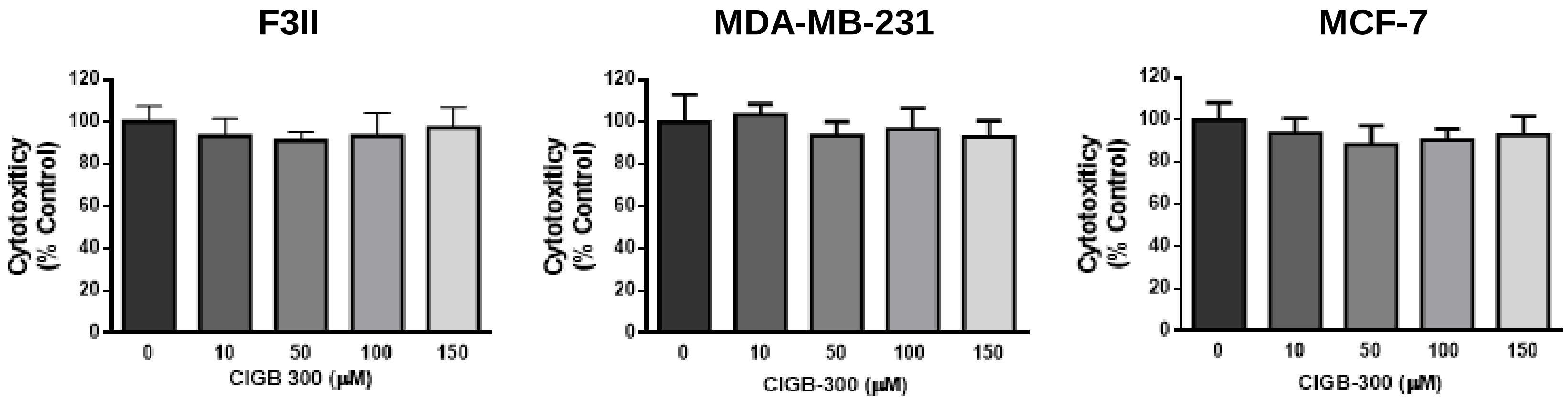

Supplemental Figure 1: Lack of cytotoxicity in breast cancer cells after short-term exposure to CIGB-300. F3II, MDA-MB-231 and MCF-7 cells were incubated with CIGB-300 for 24 h at different concentrations. Cell viability was determined by a colorimetric method. Each point represents the average of six independent measurements, each done in triplicate with the standard error mean. *p<0,05; **p<0,01 ANOVA and Dunnett post-test.
